# Novel, complex configurations of the *MARCHF6* repeat expansion associated with progressive myoclonic epilepsy and familial adult myoclonic epilepsy

**DOI:** 10.1093/braincomms/fcaf433

**Published:** 2025-11-03

**Authors:** Mark F Bennett, Mark A Corbett, Thessa Kroes, Laura Canafoglia, Karen L Oliver, Jillian M Cameron, Neblina Sikta, Jacob Munro, Liam G Fearnley, Kristina Ibañez, Arianna Tucci, Sanjay M Sisodiya, Michael S Hildebrand, Ingrid E Scheffer, Carolina Courage, Anna-Elina Lehesjoki, Loretta Giuliano, Giuseppe Didato, Silvana Franceschetti, Jozef Gecz, Samuel F Berkovic, Melanie Bahlo

**Affiliations:** Population Health and Immunity Division, The Walter and Eliza Hall Institute of Medical Research, Parkville, Victoria 3052, Australia; Department of Medical Biology, The University of Melbourne, Parkville, Victoria 3052, Australia; Epilepsy Research Centre, Department of Medicine, The University of Melbourne, Austin Health, Heidelberg, Victoria 3084, Australia; Robinson Research Institute & Adelaide Medical School, The University of Adelaide, Adelaide, South Australia 5005, Australia; Robinson Research Institute & Adelaide Medical School, The University of Adelaide, Adelaide, South Australia 5005, Australia; Department of Diagnostic and Technology, Fondazione IRCCS Istituto Neurologico Carlo Besta, Milan 20133, Italy; Population Health and Immunity Division, The Walter and Eliza Hall Institute of Medical Research, Parkville, Victoria 3052, Australia; Epilepsy Research Centre, Department of Medicine, The University of Melbourne, Austin Health, Heidelberg, Victoria 3084, Australia; Epilepsy Research Centre, Department of Medicine, The University of Melbourne, Austin Health, Heidelberg, Victoria 3084, Australia; Epilepsy Research Centre, Department of Medicine, The University of Melbourne, Austin Health, Heidelberg, Victoria 3084, Australia; Population Health and Immunity Division, The Walter and Eliza Hall Institute of Medical Research, Parkville, Victoria 3052, Australia; Department of Medical Biology, The University of Melbourne, Parkville, Victoria 3052, Australia; Population Health and Immunity Division, The Walter and Eliza Hall Institute of Medical Research, Parkville, Victoria 3052, Australia; Department of Medical Biology, The University of Melbourne, Parkville, Victoria 3052, Australia; Murdoch Children’s Research Institute, Parkville, Victoria 3052, Australia; William Harvey Research Institute, Queen Mary University of London, London EC1M 6BQ, UK; William Harvey Research Institute, Queen Mary University of London, London EC1M 6BQ, UK; Department of Clinical and Experimental Epilepsy, UCL Queen Square Institute of Neurology, Queen Square, London WC1N 3BG, UK; Chalfont Centre for Epilepsy, Chalfont St Peter SL9 0RJ, UK; Epilepsy Research Centre, Department of Medicine, The University of Melbourne, Austin Health, Heidelberg, Victoria 3084, Australia; Murdoch Children’s Research Institute, Parkville, Victoria 3052, Australia; Epilepsy Research Centre, Department of Medicine, The University of Melbourne, Austin Health, Heidelberg, Victoria 3084, Australia; Murdoch Children’s Research Institute, Parkville, Victoria 3052, Australia; Bladin-Berkovic Comprehensive Epilepsy Program, Department of Neurology, Austin Health, Heidelberg, Victoria 3084, Australia; Department of Paediatrics, The University of Melbourne, Royal Children’s Hospital, Parkville, Victoria 3052, Australia; The Florey Institute, Parkville, Victoria 3052, Australia; Folkhälsan Research Center, Helsinki 00290, Finland; Department of Medical and Clinical Genetics, Medicum, University of Helsinki, Helsinki 00014, Finland; Folkhälsan Research Center, Helsinki 00290, Finland; Department of Medical and Clinical Genetics, Medicum, University of Helsinki, Helsinki 00014, Finland; Department of Medical and Surgical Sciences and Advanced Technologies ‘G.F. Ingrassia’, Section of Neurosciences, University of Catania, Catania 95123, Italy; Department of Diagnostic and Technology, Fondazione IRCCS Istituto Neurologico Carlo Besta, Milan 20133, Italy; Department of Diagnostic and Technology, Fondazione IRCCS Istituto Neurologico Carlo Besta, Milan 20133, Italy; Robinson Research Institute & Adelaide Medical School, The University of Adelaide, Adelaide, South Australia 5005, Australia; South Australian Health and Medical Research Institute, Adelaide, South Australia 5000, Australia; Epilepsy Research Centre, Department of Medicine, The University of Melbourne, Austin Health, Heidelberg, Victoria 3084, Australia; Bladin-Berkovic Comprehensive Epilepsy Program, Department of Neurology, Austin Health, Heidelberg, Victoria 3084, Australia; Population Health and Immunity Division, The Walter and Eliza Hall Institute of Medical Research, Parkville, Victoria 3052, Australia; Department of Medical Biology, The University of Melbourne, Parkville, Victoria 3052, Australia

**Keywords:** epilepsy genetics, FAME3, short tandem repeats, pentanucleotide repeat disorders, whole genome sequencing

## Abstract

Repeat expansions are a known cause of progressive myoclonic epilepsy (PME) and familial adult myoclonic epilepsy (FAME). We hypothesized that PME and FAME may have an overlapping phenotypic spectrum and searched for pathogenic repeat expansions in 18 individuals from 15 families with later-onset PME or FAME. We generated whole genome sequencing data by short-read sequencing and searched for known and novel repeat expansions. No known, pathogenic repeat expansions were identified. Instead, we discovered a novel TTGTA expansion in the gene *MARCHF6* at the same location as the known, pathogenic FAME3 expansion in a PME family. Targeted long-read sequencing of this locus revealed a large, complex repeat structure harbouring an expansion of the pathogenic TTTCA repeat that causes FAME, surrounded by TTTTA and TTGTA expansions. Motivated by this discovery, we developed a new bioinformatic approach, mixSTR, to search for evidence of such complex expansions and discovered an additional novel configuration of the FAME3 expansion containing hidden pathogenic TTTCA expansions embedded within a TTTTA expansion in a second family clinically diagnosed with FAME. Both families had initially tested negative for the FAME3 expansion with standard RP-PCR and short-read genome sequencing analysis. We searched large epilepsy and population cohorts but did not identify any additional new individuals with complex FAME3 expansions. These findings have two important implications. Firstly, known repeat expansion loci with unusual repeat expansions, even if not known to be pathogenic, warrant further investigation as they may contain hidden pathogenic repeat expansions. Secondly, they provide molecular support for the clinical idea that PME and FAME have an overlapping phenotypic spectrum, and that the known FAME repeat expansions should be part of the diagnostic assessment of unsolved PMEs.

## Teaser

Bennett *et al*. discovered hidden complex repeat expansions in the gene *MARCHF6* in two families with rare forms of epilepsy. These expansions were missed by current repeat expansion testing but were revealed by a new analysis approach. The findings suggest that these two forms of epilepsy have an overlapping phenotype.

## Graphical Abstract

**Graphical Abstract:**
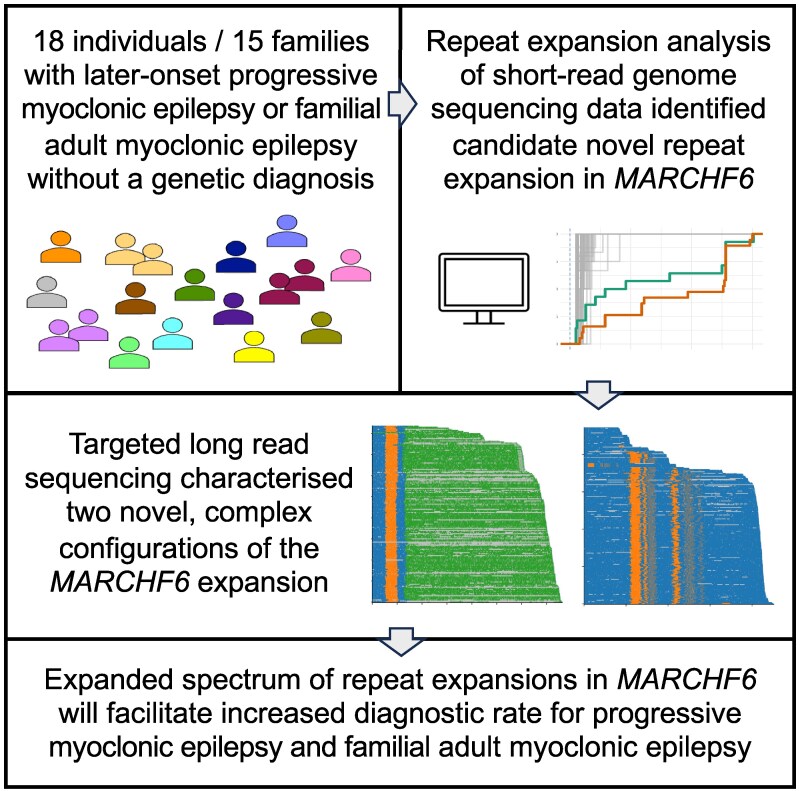

## Introduction

Progressive myoclonic epilepsies (PMEs) are a heterogeneous group of neurodegenerative diseases.^1^ PME is characterized by the presence of myoclonus, tonic-clonic seizures and progressive neurological deterioration.^1^ Symptoms typically onset in adolescence and respond poorly to treatment. Amongst the >40 genetic causes of PME, Unverricht-Lundborg disease is the most frequently observed form^2^ and is typically caused by the recessive inheritance of a 12 base pair (bp) repeat expansion (RE) in the promoter region of the gene *CSTB*.^3^ REs are large, unstable expansions of short tandem repeats (STRs). REs are known to cause at least 60 human diseases, primarily neurological, including eight forms of epilepsy.^4^ In addition to Unverricht-Lundborg disease, seven REs have been identified as the cause of familial adult myoclonic epilepsy (FAME),^5-9^ an autosomal-dominant, adult-onset epilepsy characterized by a cortical myoclonic tremor, with seizures often, but not always, presenting later in life and with little or no progression.^10^

FAME is caused by the insertion of an intronic pathogenic TTTCA expansion in at least seven different genes, causing FAME1-4 and FAME6-8.^5-9^ The FAME expansions all have similar, complex repeat structures containing expansions of multiple repeat motifs, a characteristic also shared by other pentanucleotide repeat disorders.^11^ Most FAMEs are observed with adjacent TTTCA and TTTTA expansions;^5-9^ however, for FAME6, FAME7, and in a small number of FAME1 families, the pathogenic TTTCA expansion is surrounded by TTTTA repeats.^5^

We studied 15 families with later-onset PME or suspected FAME. We have previously noted that historically FAME families were likely conflated with PMEs^12^ and hypothesized that PME and FAME lie on a phenotypic spectrum, and FAME-like expansions may cause milder, later-onset PME.^13^ We generated short-read whole genome sequencing (WGS) data and searched for known and novel REs, then performed molecular validation and long-read sequencing to characterize novel, complex configurations of the FAME3 RE identified in two families.

## Materials and methods

### Short-read genome sequencing

We studied 15 families with a clinical diagnosis of later-onset PME or suspected FAME. The study was approved by the Human Research Ethics Committees of Austin Health (Project No. H2007/02961) and the Walter and Eliza Hall Institute of Medical Research (Project No. G20/01). Informed consent was obtained for all participants.

DNA was extracted from whole blood for WGS with 30× target coverage. Linked-read genomic sequencing was performed for 15 individuals (nine females, six males) from 13 families and three individuals (two females, one male) from two families were sequenced using a PCR-free protocol on Illumina NovaSeq. The 10× linked-read technology generates short-read sequencing data with long-range information. Paired 151 bp reads include a 16 bp barcode representing the larger DNA fragment they originate from. We removed the 16 bp barcodes, discarding the linked read information and aligned the resulting 151 and 135 bp paired reads and the 151 bp paired reads Illumina NovaSeq WGS data to the hg38 reference genome with BWA-MEM v0.7.17-r1188^14^ and then performed duplicate marking and base quality score recalibration using GATK v4.0.11.0,^15^ following GATK best practices.

### Short-read RE analysis

We interrogated the aligned WGS data for known and novel disease-causing REs, as previously described.^16^ Disease-causing REs were targeted using ExpansionHunter^17^ (v2.5.5 and v5.0.0) and exSTRa^18^ (v0.90.0 with Bio-STR-exSTRa v1.0.3) with default settings, using the database available at https://github.com/bahlolab/RepeatExpansionDatabase (accessed 3 October 2023). ExpansionHunter v5.0.0 output was visualized using REViewer (v0.2.7).^19^

A genome-wide search for novel REs was performed using ExpansionHunter Denovo (EHDN)^20^ (v0.9.0) with the additional ‘--max-irr-mapq 60’ command line option. Specifying this parameter forces EHDN to search all reads (as the maximum MAPQ value is 60) for evidence of REs, as we have found that expansions of some motifs map to the genome with MAPQ > 40 (default parameter value) and could be overlooked. Post-processing of EHDN profiles was performed independently for samples in different sequencing batches, including additional samples from each sequencing batch with different diagnoses as ‘controls’. Targeted analysis to follow-up the novel TTGTA expansion identified by EHDN was performed for 169 inhouse WGS samples from individuals with epilepsy or their family members and 3202 WGS samples from the 1000 Genomes^21^ as described above, with modified input files for the FAME3 locus, replacing the TTTCA repeat motif with TTGTA (available on request).

### Repeat-primed and long-range PCR

Repeat-primed PCR (RP-PCR) was performed as previously described to test for the TTTCA motif at the FAME3 locus.^7^ TTGTA expansions were identified by RP-PCR by substituting the TTTCA repeat primer with RP-FAME3-TTGTA: 5′ TACGCATCCCAGTTTGAGACGTTGTATTGTATTGTATTGTATTGTATTGTA 3′ and using the same PCR buffer and cycling conditions as previously described.^7^

Long-range PCR (LR-PCR) was performed with 50 ng of genomic DNA in LongAmp® HotStart Taq master mix (New England Biolabs #M0533) according to the manufacturer’s conditions except for the following modifications: 5 mM dGTP and dCTP, 15 mM dATP and dTTP, 3 mM MgSO_4_ and 0.5 μM of each primer pair per reaction. Primer sequences were: LR-PCR_FAME3_F2: 5′ CACTTAAAGGAAAAGGGAGGGTTATAGAGGA 3′ and LR-PCR_FAME3_R3: 5′ CGCACGGTTGATGTGTTTGTAACAT 3′. PCR cycling conditions were 94°C for 2 min, followed by 30 cycles of 94°C for 30 s, 60°C for 30 s and 62.5°C for 10 min, then a final replication step of 65°C for 10 min.

### Targeted long-read sequencing

Family A: LR-PCR products were sequenced in an Oxford Nanopore R9.4.1 minION flow cell using the protocol provided by the manufacturer (Native barcoding amplicons using the EXP-NBD104, EXP-NBD114 and SQK-LSK109 kits and protocol version: NBA_9093_v109_revC_12Nov2019). Sequence data were collected using a minION Mk1B with MinKNOW control software v23.04.5 (III-1) and v23.07.5 (II-4), and bases called with Dorado v7.3.11 using the super accurate model: dna_r9.4.1_450bps_sup.

Family B: LR-PCR products were sequenced in an Oxford Nanopore R10.4.1 Flongle flow cell using the protocol provided by the manufacturer (Native barcoding amplicons using the SQK-NBD114-24 kit and protocol version: NBA_9168_V114_REVL_15SEP2022). Sequence data were collected using a minION Mk1B with MinKNOW control software v23.11.3 and bases called with Dorado v7.3.11 using the super accurate model dna_r10.4.1_e8.2_400bps_sup@v4.3.0. Reads where large tracts of the TTTCA and TTTTA motifs were incorrectly called as TTCCA by Dorado were excluded from the analysis. Incorrectly called reads were identified by visual inspection and due to their directional bias mapping only to the reverse chromosome strand.

Sequence data were mapped to the hs38d1 build of the human genome (available from https://ftp.ncbi.nlm.nih.gov/genomes/all/GCA/000/001/405/GCA_000001405.15_GRCh38/seqs_for_alignment_pipelines.ucsc_ids/GCA_000001405.15_GRCh38_no_alt_plus_hs38d1_analysis_set.fna.gz) using minimap2^22^ with recommended parameters for nanopore sequencing.

Mapped sequences were viewed using the Integrative Genome Viewer. Quantification of TTTTA, TTTCA and TTGTA motif counts was made using PacBio repeat analysis tools https://github.com/PacificBiosciences/apps-scripts/tree/master/RepeatAnalysisTools. Mapped repeat containing reads were extracted from the region chr5:10356339-10356411 (hg38) using *extractRegion.py*. Repeat counts were made using *countMotifs.py*, then repeats with total lengths <800 bp were filtered out to remove the wild type alleles, summarize motif count distributions and create waterfall plots.

### Screening exome sequencing cohorts for TTGTA expansions

We screened 151 bp paired-end whole exome sequencing (WES) data for 1616 individuals (861 females, 755 males) with a variety of epilepsy subtypes recruited in Australia, sequenced through the Epi25 Collaborative as previously described.^23^ The FAME3 locus is not captured by the WES. We have previously demonstrated that off-target WES data can be informative for non-coding REs in some samples.^24^ Therefore, we searched for samples with off-target reads that may provide evidence of large TTGTA expansions anywhere in their genome. Samples with at least three read pairs where both reads were determined by superSTR^25^ (v1.0.1) to have ≧125 bp of TTGTA repeats or identified by EHDN as paired in-repeat reads (>90% TTGTA repeats), were followed up with targeted analysis. Long-range PCR was employed to search for the presence of an expansion at the FAME3 locus and, if an expansion was found, targeted long-read sequencing was performed as described above.

### Searching genome sequencing cohorts for complex FAME3 expansion

We searched 3202 control individuals (1604 females, 1598 males) with high-coverage WGS from the 1000 Genomes Project cohort^21^ and 2209 participants (1161 females, 1048 males) with ‘inherited epilepsy syndrome’ as their normalized disease subgroup (Rare Disease programme) from the 100 000 Genomes Project in the UK^26^ for TTGTA expansions at the FAME3 locus using ExpansionHunter as described above. STR genotypes of known REs for 19 421 WGS samples were downloaded from the Genome Aggregation Database (gnomAD) dataset (v2022-01-20).^27^ The gnomAD genotypes were generated using an optimized version of ExpansionHunter (v5.0.0), with a custom script that allows for different repeat motifs to be identified for pentamer expansions.

### Searching for paired reads containing mixed-motif STR expansions

Bioinformatic RE detection methods rely on reads that align uniquely to the flanking DNA surrounding the repeat locus, as reads containing pure repetitive sequences are often misaligned or unaligned. Therefore, they cannot assess the interior of large, complex expansions. Motivated by our discovery of the complex configurations of the FAME3 REs, we developed a new bioinformatic approach, called mixSTR, to search for paired reads containing mixed-motif expansions that support the presence of complex repeat configurations, containing adjacent expansions of multiple different repeat motifs.

mixSTR is a python script (available at https://github.com/bahlolab/mixSTR) that processes output generated by superSTR^25^ to search for evidence of mixed-motif expansions, e.g. (TTTTA)_exp_-(TTTCA)_exp_, the structure of most observed FAME expansions. mixSTR identifies mixed-motif expansion read pairs by filtering for read pairs where both reads consist (nearly) entirely of pure repetitive sequence (default: >125 bp repeat sequence in both reads in the pair) and where all specified target repeat motifs exceeding a minimum threshold of repeat sequence (default: 50 bp across the pair). We used mixSTR to screen WGS samples to identify mixed-motif read pairs containing both TTTTA and TTTCA expansions, the characteristic signature of reported FAME expansions.

## Results

### Short-read RE analysis identifies TTGTA expansion at the FAME3 locus

**Figure 1:**
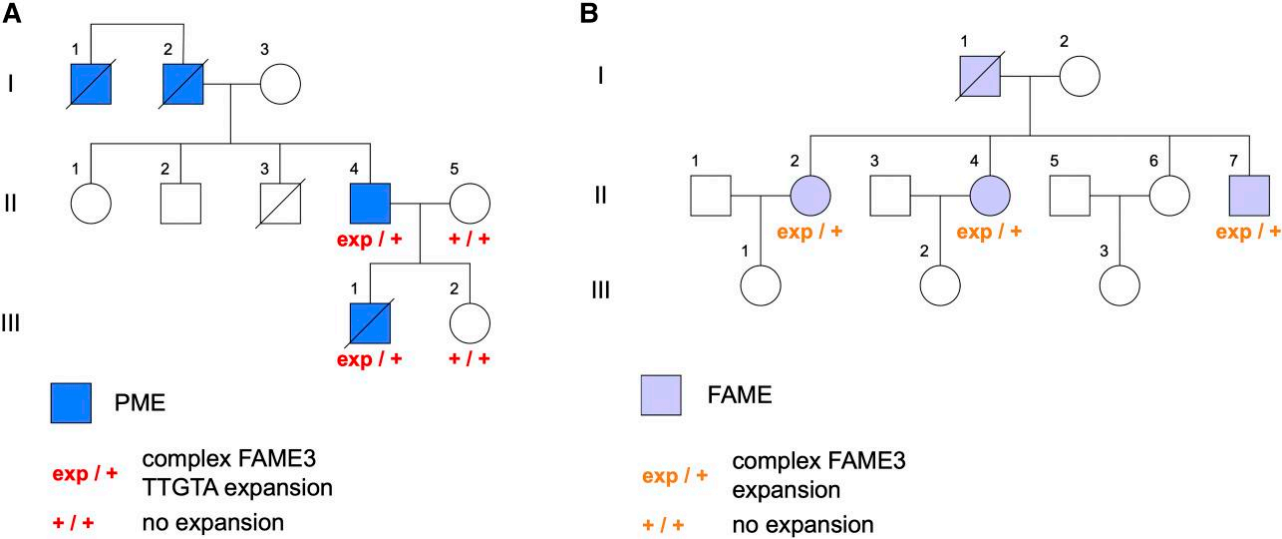
**Family pedigrees and segregation results. (A)** Late-onset PME family. **(B)** FAME family.

**Figure 2:**
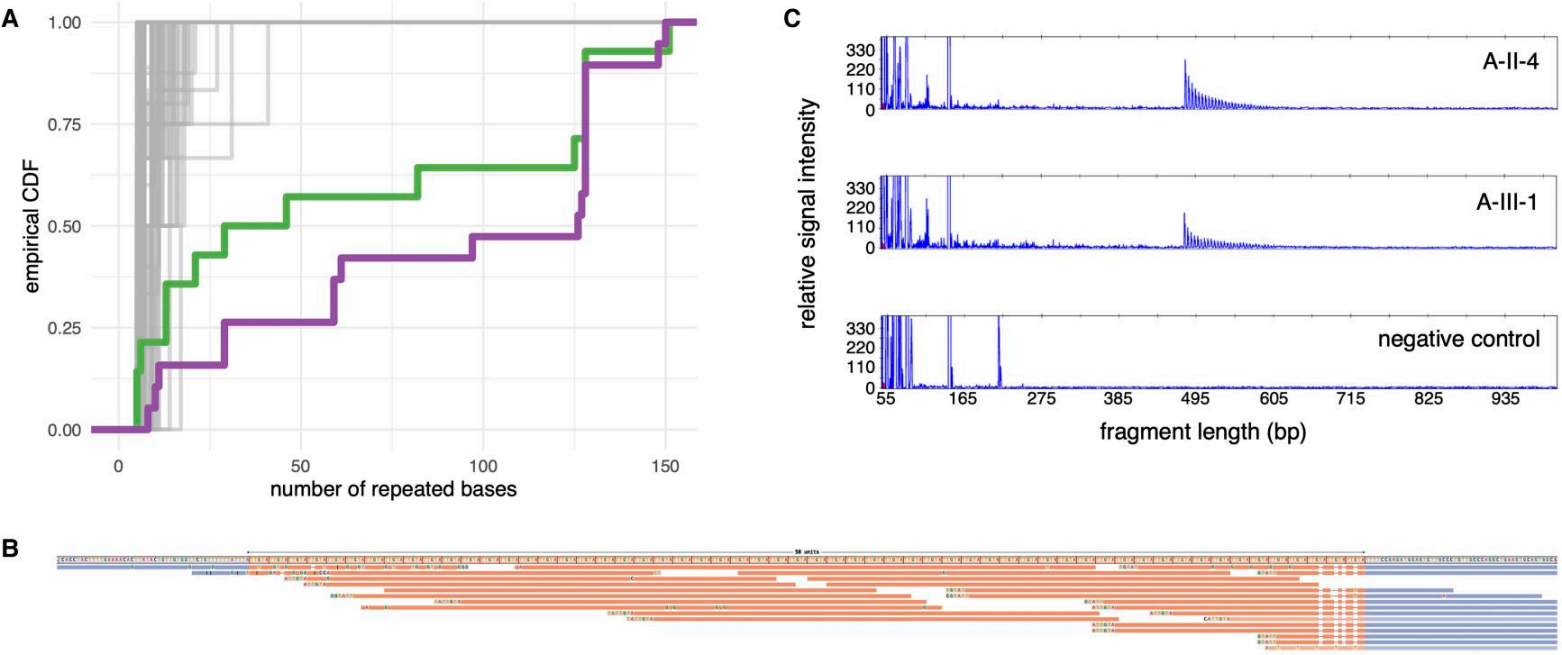
**TTGTA expansion at FAME3 locus in the gene *MARCHF6*. (A)** exSTRa empirical cumulative distribution function showing the distribution of TTGTA repeated bases in WGS data at FAME3 locus. Father (A-II-4) and son (A-III-1) are highlighted in green and purple, respectively, with other samples from the epilepsy WGS cohort in grey. Both father and son are observed to be outliers in the distribution of base pairs containing TTGTA repeat sequence. (**B**) REViewer image of ExpansionHunter output, cropped to only show the larger allele. ExpansionHunter predicts a large TTGTA expansion at this locus, with size >50 repeats. Reads containing large TTGTA repeats are confidently anchored only to the right flank. (The two reads overlapping the left flank align poorly, with numerous sequencing errors.) (**C**) RP-PCR confirmation of TTGTA expansion.

Analysis of WGS data for 18 individuals from 15 families was performed to search for known, disease-causing REs. No pathogenic REs were identified. We searched genome-wide for REs using ExpansionHunter Denovo (EHDN) and identified a novel TTGTA expansion in the gene *MARCHF6* at the known FAME3 locus in a father–son duo with later-onset PME (Family A; Fig. 1A). The father–son duo had previously undergone whole exome sequencing analysis for variants in known PME genes without achieving a genetic diagnosis.^13^ This TTGTA expansion was confirmed by targeted bioinformatic RE analysis and RP-PCR (Fig. 2). The unaffected mother (A-II-5) and daughter (A-III-2) do not carry the TTGTA expansion (Fig. 1A). RP-PCR testing did not identify the known pathogenic TTTCA FAME expansions in the father or son (Supplementary Fig. 1A and B).

### Long-read sequencing reveals complex TTGTA FAME3 repeat structure

**Figure 3:**
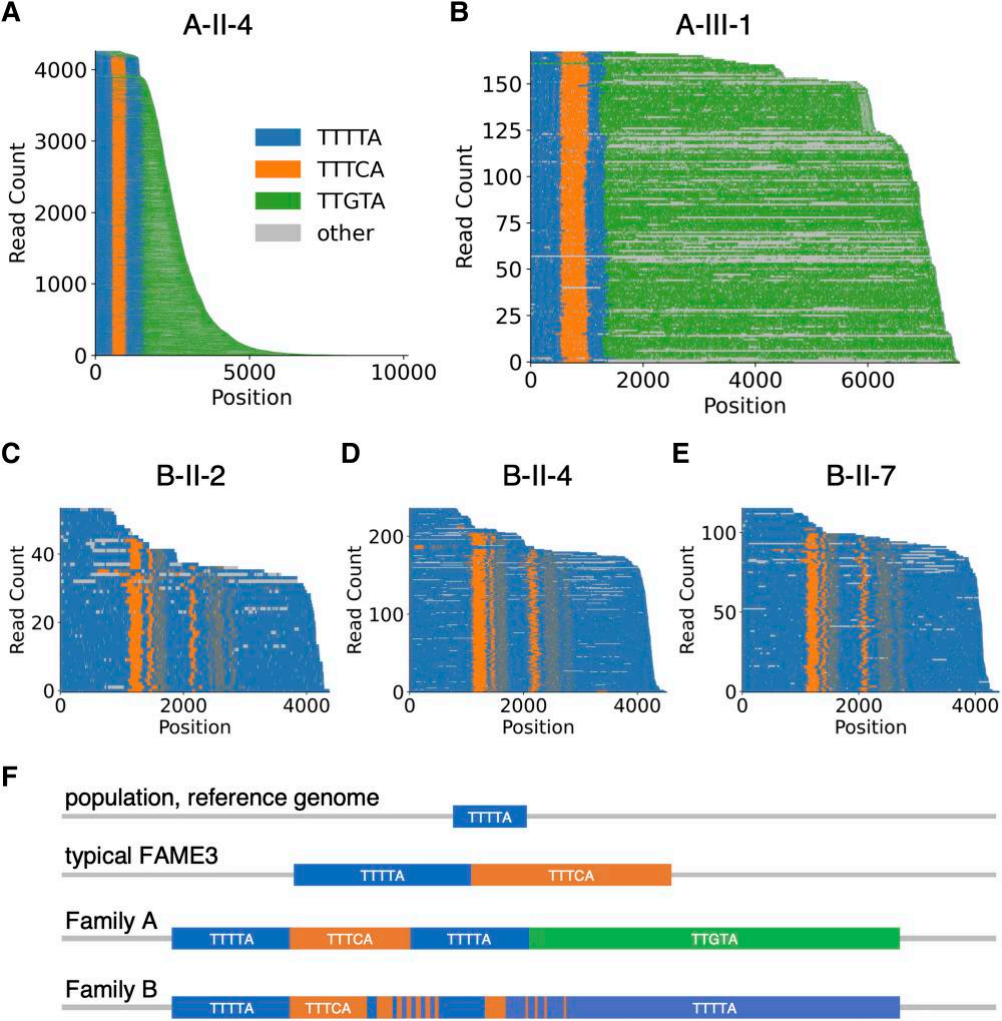
**Complex RE structures at FAME3 locus revealed by long-read sequencing.** Waterfall plots of FAME3 locus from nanopore sequencing of long-range PCR products for **(A)** father (A-II-4), and **(B)** son (A-III-1) from Family A with later-onset PME; and affected siblings with FAME **(C)** B-II-2, **(D)** B-II-4, **(E)** B-II-7 from Family B. **(F)** Conceptual illustration of alternative repeat configurations observed at the FAME3 locus. Short, pure TTTTA repeats are present in the reference genome and most of the population. Typical FAME3 families have adjacent TTTTA and TTTCA expansions. The two families identified in this study have complex repeat configurations containing pathogenic TTTCA expansions surrounded by expansions of other pentamer motifs.

Targeted long-read sequencing of long-range PCR products revealed a large, complex repeat structure at the FAME3 locus, present in both the father and son in Family A (Fig. 3A and B). In addition to the TTTTA and TTGTA expansions identified by analysis of short-read WGS, a TTTCA expansion was identified buried deep within the following complex repeat structure, (TTTTA)_exp_-(TTTCA)_exp_-(TTTTA)_exp_-(TTGTA)_exp_. All known FAMEs are associated with pathogenic TTTCA expansions.^5-9^ The buried TTTCA expansion is not directly detectable by RP-PCR nor by short-read RE methods, which rely on paired reads uniquely mapped to flanking DNA and can only penetrate a limited distance into the repeat structure.

The size of the TTTTA and TTTCA expansions appears to be stable across all long-sequencing reads in both the father and son at around 500 bp, representing 100 copies of these pentanucleotide repeats. In contrast, there is notable variation in the size of the TTGTA expansion (Fig. 3A-B). This variation may be due to PCR bias or could represent true mosaicism of the TTGTA repeat. Genomic DNA of sufficient quality and quantity was unavailable to generate long-read sequencing using an amplification-free method to confirm this.

### Discovery of second complex FAME3 repeat structure

We developed a new bioinformatic approach, mixSTR, to screen short-read WGS for paired reads with expansions of multiple repeat motifs that suggest the presence of complex REs (see Methods for details). We used mixSTR to search for mixed-motif pairs containing TTTCA and TTTTA expansions, the characteristic signature of the FAME REs, in the WGS data from the remaining 16 individuals with later-onset PME or suspected FAME. We identified one individual with suspected FAME (Family B, II-2; Fig. 1B) who had many TTTTA + TTTCA mixed-motif read pairs. Upon closer review, their EHDN output also indicated the presence of a TTTTA expansion at the FAME3/*MARCHF6* locus. TTTTA expansions at the FAME3 locus are relatively common in the population (∼0.6% of individuals in the gnomAD dataset have TTTTA expansions >150 bp); therefore, this finding was not an outlier and was initially dismissed. However, motivated by the high number of TTTTA + TTTCA mixed-motif read pairs identified by mixSTR, we performed long-read sequencing of the FAME3 locus to investigate.

Targeted long-read sequencing of long-range PCR products for B-II-2 and two affected siblings (B-II-4 and B-II-7; Fig. 1B) revealed a large, complex repeat structure at the FAME3 locus containing TTTTA and TTTCA expansions in all three siblings (Fig. 3C–E). A consistent repeat structure is observed in all siblings, with a total size of ∼4.5 kb, consisting mainly of TTTTA repeats but also containing embedded TTTCA expansions buried within the TTTTA expansion, and some segments containing TTTTATTTTATTTCA repeats, a 15 bp repeat motif that is a combination of these two pentamer motifs. Previous RP-PCR testing of B-II-2 was negative for a TTTCA expansion (Supplementary Fig. 1C).

### Haplotype analysis

We compared the haplotypes in the region surrounding the FAME3/*MARCHF6* locus for Family A, Family B and the haplotype reported for two families in the original discovery of FAME3 (see Supplementary Fig. 5 in Florian *et al*.^7^). Families A and B both have the same shared genetic markers as the FAME3 haplotype previously reported.^7^ However, LDhap^28^ determines that this haplotype is also shared by 8.7% of 1000 Genomes families with European ancestry. Therefore, while both families are consistent with a shared FAME3 founder haplotype, we cannot determine this with confidence due to the high background rate of sharing of this haplotype in Europeans. Families A and B are both from Italy; however, Family A is from the far north of the peninsula, while Family B is from the far south and the affected father (B-I-1) came from North Africa.

### Screening additional cohorts identifies pure TTGTA FAME3 locus expansions

We searched several large epilepsy and population cohorts for additional individuals carrying the novel, complex TTGTA FAME3 locus expansion. Searching for examples of the second complex FAME3 configuration observed in Family B is more challenging, as this configuration is indistinguishable using short-read data from pure (benign) TTTTA expansions that are relatively common, except for the possible presence of mixed-motif TTTTA + TTTCA read pairs. However, we identified a high background rate of mixed-motif TTTTA + TTTCA read pairs in the 1000 Genomes WGS population cohort. The origin of these reads cannot be established without performing follow-up long-read sequencing, which was not possible for this cohort. Therefore, we focused on searching for TTGTA expansions at the FAME3 locus that could reveal the presence of the complex configuration identified in Family A.

No TTGTA FAME3 expansions were identified in 169 WGS samples from an inhouse epilepsy cohort and 3202 samples from the 1000 Genomes Project with publicly available WGS data.^21^ We screened 1616 individuals with epilepsy and available WES data for evidence of large TTGTA expansions somewhere in their genome. The FAME3 locus is not targeted by WES but off-target reads may be informative, as this strategy was previously shown to be successful for some individuals with the non-coding RE that causes cerebellar ataxia with neuropathy and vestibular areflexia syndrome (CANVAS).^24^ Ten individuals identified with three or more read pairs containing (nearly) pure TTGTA repeat sequences were investigated using long-range PCR and targeted nanopore sequencing. One of these individuals had a large TTGTA FAME3 expansion (Supplementary Fig. 2B). Unlike the complex RE identified in Family A, long-read sequencing revealed a pure TTGTA expansion exceeding 5 kb. This individual had epilepsy with eyelid myoclonus, which was paternally inherited.

We interrogated WGS data for 2209 individuals with epilepsy from the 100 000 Genomes Project in the UK and identified one TTGTA expansion, predicted to have >40 TTGTA repeats at the FAME3 locus. This individual had a clinical diagnosis of focal epilepsy with a genetic diagnosis of a likely pathogenic variant in *KCNT1* that matches their phenotype and no clinical features indicative of FAME or PME. We visualized sequencing reads using REViewer and determined that reads containing TTGTA expansions are aligned to both left and right flanking regions (Supplementary Fig. 2A). Short-read data do not permit characterization of the interior repeat structure; however, given that TTGTA repeats are anchored to both left and right flanks, we infer that this individual likely has a pure TTGTA expansion, like the individual we characterized with long-read sequencing (Supplementary Fig. 2B).

### Prevalence of TTGTA expansions at other FAME loci

STR calls for known, disease-causing loci, including FAME3, are available for 19 241 individuals from gnomAD. No individuals are reported with TTGTA repeats at the FAME3 locus; however, there are 11 individuals with TTGTA repeats reported at other FAME loci. Two individuals have FAME1 TTGTA expansions with estimated sizes of 42 and 47 repeats. For FAME2, there is one normal size (11 repeats) and eight TTGTA expansions with an estimated size between 39 and 78 repeats. For each of these, we manually reviewed sequencing data through the gnomAD web interface. Sequencing reads containing TTGTA repeats are anchored to both left and right flanks for 10 of the 11 alleles, suggesting that these alleles are likely pure TTGTA expansions. One FAME2 allele has a different configuration, with TTGTA repeats anchored to the left flank only and TTTTA repeats anchored to the right flank. This allele has a more complex configuration of 5′-(TTTTA)_exp_-(N)-(TTGTA)_exp_-3’′ in the context of *STARD7*, which lies on the reverse strand. We could not fully resolve the repeat structure, as it is not possible to obtain more information about the interior regions of the repeat using short-read sequencing data alone.

### Clinical phenotyping of family A with later-onset PME

The proband (A-II-4) was a 65-year-old man who had his first tonic-clonic seizure at age 19 years. After the first seizure, he was treated with the combination of phenobarbital and phenytoin; from the age of 23 until 40 years, he had sporadic seizures (2–5/year) during sleep, characterized by diffuse tonic contraction often preceded in the last 2 h by spontaneous myoclonic jerks (at progressively increasing frequency). At the age of 40, after the addition of carbamazepine, he showed a worsening of the frequency of the seizures during sleep and the appearance of seizures also during wakefulness, characterized by massive myoclonic jerks. At the age of 41, he was diagnosed with PME, and therapy was changed with the addition of valproate and the discontinuation of carbamazepine and phenytoin. Although the myoclonic jerks improved, he still had sporadic tonic seizures during sleep until the age of 55 and occasional falls during the day due to jerks at lower limbs. Spontaneous and movement-induced myoclonic jerks were noted from age 24 years and slightly worsened over the years. First examination (65 years) revealed dysarthria but no other cerebellar signs. Action myoclonus was of moderate severity, evident especially in the upper limbs and face, and there was an ongoing cortical tremor. In later years, his gait worsened due to the appearance of cerebellar and extrapyramidal signs, with moderate degrees of bradykinesia. EEG showed a normal background with diffuse spikes sometimes associated with jerks. There was photosensitivity at 5–20 Hz stimulation. Simultaneous EMG recording during voluntary motor activation showed short EMG bursts, consistent with myoclonus, repeated with high frequency (Supplementary Fig. 3A). Overall, the EEG-EMG features were suggestive of a form of classic PME. MRI (performed at the age of 70) showed mild cerebellar atrophy (not observed in previous exams). Somatosensory evoked potential amplitude was increased, and C reflex was enhanced. MMSE showed a score of 21, indicating a mild mental impairment. According to family members, there was a slow and progressive decline in cognition and attention, starting at about age 40.

His 40-year-old son (A-III-1) had tonic-clonic seizures, often occurring during sleep, from age 27 years, rare atypical absences from age 35 years and myoclonic jerks with falls from age 38. He had a personality disorder and complained about memory deficits. EEG showed a normal background with multifocal (bilateral fronto-temporal) and diffuse spikes. Simultaneous EMG recording documented multifocal and action myoclonus facilitated by intermittent light stimulation, with similar characteristics to the father (Supplementary Fig. 3B). His response to medication, including valproate, was unsatisfactory. The neurological examination revealed no neurological deficits, but action myoclonus in all four limbs and a cortical tremor. A back-averaging analysis supported a cortical origin of the myoclonus, as well as a slight increase in the amplitude of the somatosensory evoked potentials and the presence of enhanced C reflex response. He died a few months after our observation due to an accidental death unrelated to the epilepsy. The clinical pattern suggested the same disease as the father (PME).

Both father and son were included as part of an Italian PME collection, as unsolved cases with classification ‘PME with dementia’ (father) and ‘late-onset PME’ (son).^29^

### Clinical phenotyping of Family B with FAME

The proband (B-II-2) presented with repetitive myoclonic seizures affecting the upper limbs and a persistent hand tremor at the age of 26. She had rare tonic-clonic seizures beginning at the age of 28, preceded by spontaneous jerks. Neurological examination revealed mild hyperreflexia, and she had a writing-related dystonia of the right upper limb. Her EEG showed normal background activity, but there were bilateral spike and polyspike discharges at 4–5 Hz. Somatosensory evoked potentials were exaggerated. Her MRI showed mild cerebellar atrophy.

Her mother was from Sicily and unaffected, her father was from Morocco and had seizures, but details were unavailable. She had one sister (B-II-4) who developed tonic-clonic seizures and bilateral tremor of the hands and facial muscles at the age of 26, and a younger brother (B-II-7) who, at the age of 22, developed rare seizures and tremors of the hands and peri-orbital muscles.

Seizures in the family generally responded to anti-epileptic medication, but tremor and myoclonus persisted. The cognitive profile of the affected individuals ranged from normal cognitive function in the younger brother to mild deficits in memory and attention domains in the older sisters. FAME was suspected clinically.

## Discussion

We generated short-read WGS data and searched for REs in 18 individuals from 15 families with later-onset PME or suspected FAME, motivated by the discovery that FAME is a RE disorder and hypothesizing that PME and FAME may lie on a phenotypic spectrum. No known disease-causing REs were detected. Searching genome-wide, we identified a novel TTGTA expansion at the known FAME3 locus. Long-read sequencing revealed a complex repeat structure, containing a large TTGTA expansion in addition to TTTTA expansions and TTTCA expansions buried within. Despite searching multiple large epilepsy and population WGS cohorts, we did not identify any additional individuals with evidence that indicated the presence of a complex TTGTA FAME3 expansion. This suggests that the expansion is likely to be rare, with a frequency of <1 in 20,000; however, we cannot rule out the existence of other complex expansions deep within the repeat that cannot be detected with short-read WGS data.

We developed a new bioinformatic approach, mixSTR, to search for evidence of mixed-motif expansions, which could indicate the presence of complex REs. Using mixSTR, we identified one individual (out of the remaining 14 families studied) with a high number of TTTTA + TTTCA mixed-motif reads, and targeted long-read sequencing revealed a different novel complex configuration of the FAME3 expansion. One limitation of this approach is that we observed a high background rate for mixed motif TTTTA + TTTCA read pairs in the 1000 Genomes population cohort. Therefore, mixSTR is likely to be most useful to search individuals suspected to have FAME or another complex RE, such as SCA37^30^ or some configurations of the CANVAS expansion;^31^ however, it has low specificity and will be less useful as a general-purpose screening tool for unascertained cohorts.

The two new alternative pathogenic FAME3 configurations expand the spectrum of repeat configurations associated with epilepsy at the FAME3 locus (Fig. 3F). Similar alternative pathogenic RE configurations at the same locus have been observed for other pentanucleotide repeat disorders. Embedded TTTGA repeats have been found at the FAME1 locus,^32^ and a large FAME1 pedigree was described where affected individuals carried large FAME-like expansions with TTTGA repeats instead of the usual pathogenic TTTCA repeats.^33^ However, the presence of TTTCA repeats in the interior of a complex structure cannot be ruled out in this family. Small TTTCA repeats, with as few as 10 copies of the pathogenic motif, have been reported to cause FAME.^34^ The minimum number of TTTCA repeats needed to be pathogenic and the mechanism by which these expansions cause disease remains unclear; however, the small TTTCA repeats reported are thought to be pathogenic as they segregate in families, with affected individuals having the distinctive clinical features associated with FAME. Pathogenic alleles with different repeat motifs and configurations have also been reported for CANVAS,^31,35-37^ a pentanucleotide repeat disorder usually caused by recessive inheritance of AAGGG expansions in the gene *RFC1*. For one alternate configuration, the pathogenic AAGGG expansions are buried, surrounded by smaller AAAGG expansions,^31^ reminiscent of the complex FAME3 expansions described in this paper.

The overlapping features of milder, later-onset forms of PME and FAME have been noted in the analysis of historical cases.^12^ Family A had clinical features consistent with a PME, because of the frequency of the seizures, and the slow but consistent worsening of the neurological condition (in the father). The electrophysiological features of Family A were also indistinguishable from those of a typical form of PME, as also shown in a previous study on FAME2.^38^ In contrast, Family B was regarded clinically as typical of FAME.

The identification of two individuals with pure TTGTA expansions at the FAME3 locus, one confirmed by long-read sequencing, and 10 individuals with inferred pure TTGTA expansions at other FAME loci in the gnomAD dataset, suggests that pure TTGTA expansions at FAME loci are likely benign. FAME, like most other RE disorders, has high penetrance. The observed prevalence of TTGTA expansions at FAME loci in population databases is larger than would be expected for a highly penetrant repeat disorder; however, we cannot rule out a more subtle disease association. It is possible that the additional TTGTA expansion contributes to modifying the phenotype in Family A, compared to other reported families with the typical FAME3 expansion.

Our data show that complex expansions with novel motifs at FAME or other pentanucleotide repeat disorders may be more prevalent than previously thought. Detecting REs of novel motifs could tag the presence of a complex, pathogenic RE that is undetectable with short-read sequencing or RP-PCR. Three individuals (A-II-4, A-III-1 and B-II-2) identified with complex FAME expansions had been screened for TTTCA expansions by RP-PCR and standard WGS analysis but tested negative. A family was recently reported with FAME1 with only a small number of TTTCA repeats, identified by WGS after initially testing negative by RP-PCR.^34^ In that case, RP-PCR testing was negative because the TTTCA repeats were too small, while in our case, the pathogenic TTTCA repeat was buried deep within a complex repeat structure. While short-read WGS cannot be used to accurately characterize the interior structure of a large, complex RE, it can flag loci that should be followed up with more in-depth bioinformatic analysis or other methods, such as targeted long-read sequencing, especially if the phenotype suggests that a repeat disorder is plausible.

Our study was motivated by the hypothesis that PME and FAME may have an overlapping disease spectrum, and our experience here suggests that the clinical and neurophysiological features do not allow definitive distinction. We identified two novel, complex configurations of the FAME3 RE in two families with PME and FAME out of 15 unsolved families studied. These findings expand the spectrum of repeat configuration associated with epilepsy at the FAME3 locus and further support the idea that PME and FAME have an overlapping phenotypic spectrum, which may lead to misdiagnoses. Genetically undiagnosed individuals with later-onset, milder PME could have FAME or FAME-like REs. The approach described in this paper could reveal similar complex repeat structures, either alternative configurations of known, pathogenic REs or novel REs that have eluded discovery and can only be revealed by long-read sequencing. We hope these findings will encourage others to search for REs in families or individuals without a genetic diagnosis where a repeat disorder is possible.

## Supplementary Material

fcaf433_Supplementary_Data

## Data Availability

Data generated for this study are available from the corresponding author on reasonable request. mixSTR is available at https://github.com/bahlolab/mixSTR.
