# Supplementary material for: Novel, complex configurations of the MARCHF6 repeat expansion associated with progressive myoclonic epilepsy and familial adult myoclonic epilepsy
Source: Brain Commun. 2025 Nov 3;7(6):fcaf433. doi: 10.1093/braincomms/fcaf433 (PMC12628750; doi:10.1093/braincomms/fcaf433)
Supplement: fcaf433_Supplementary_Data [file fcaf433_supplementary_data.pdf]

Novel, complex configurations of the *MARCHF6* repeat expansion associated with progressive myoclonic epilepsy and familial adult myoclonic epilepsy

Mark F. Bennett, Mark A. Corbett, Thessa Kroes, Laura Canafoglia, Karen L. Oliver, Jillian M. Cameron, Neblina Sikta, Jacob Munro, Liam G. Fearnley, Kristina Ibañez, Arianna Tucci, Sanjay M. Sisodiya, Michael S. Hildebrand, Ingrid E. Scheffer, Carolina Courage, Anna-Elina Lehesjoki, Loretta Giuliano, Giuseppe Didato, Silvana Franceschetti, Jozef Gecz, Samuel F. Berkovic, Melanie Bahlo

Supplementary Material

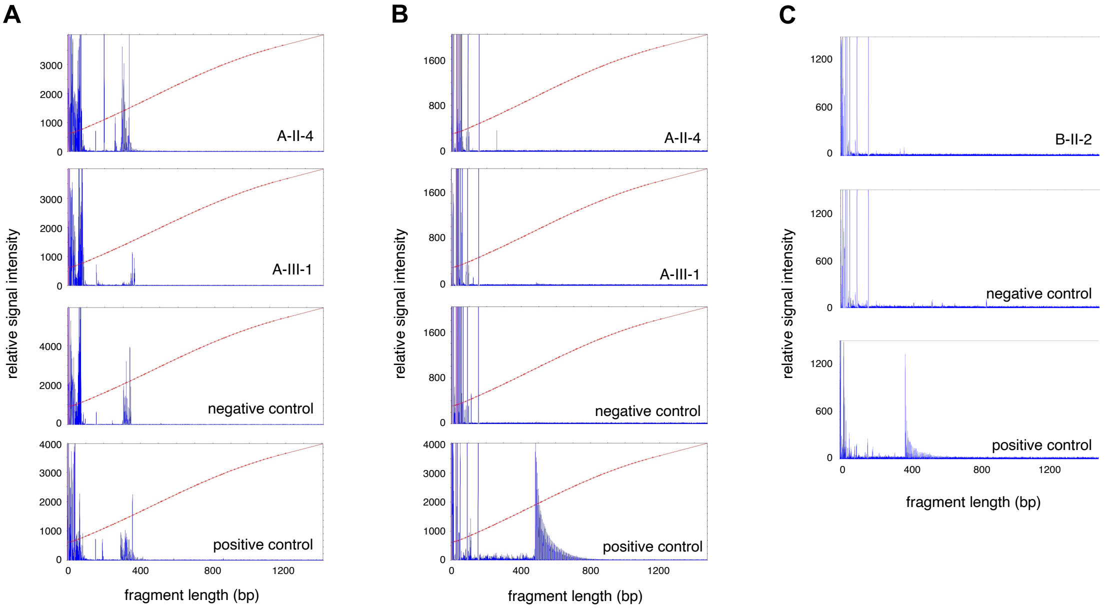

Supplementary Figure 1 Repeat-primed PCR traces at the FAME3 locus.
(A) TTTTA and **(B)** TTTCA traces for father (A-II-4) and son (A-III-1) in Family A. **(C)** TTTCA traces for B-II-2 in Family B.

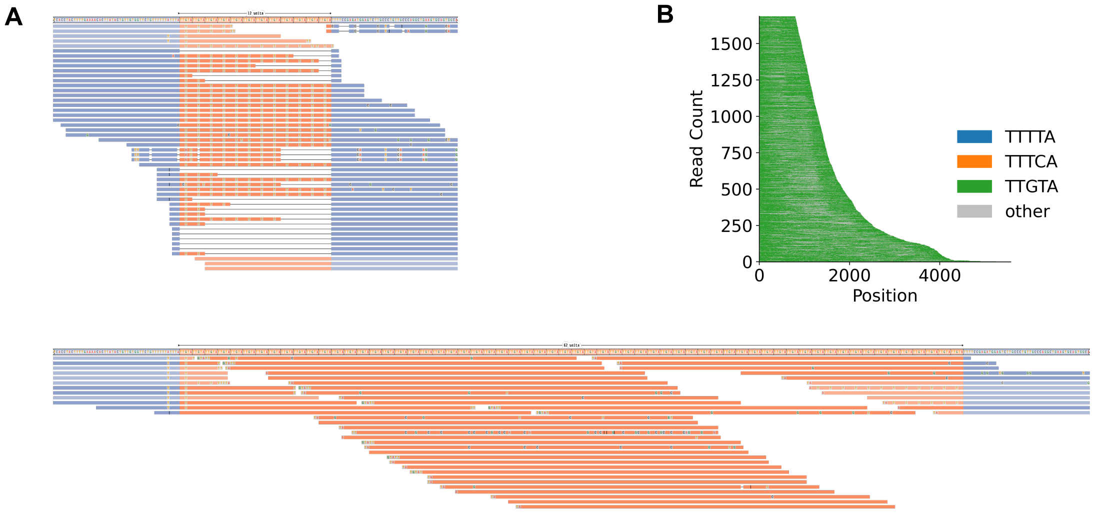

Supplementary Figure 2 Pure TTGTA expansions at the FAME3 locus.
(A) REViewer image generated from WGS for individual identified in 100,000 Genomes targeting TTGTA repeat at FAME3 locus. Larger allele shows reads containing TTGTA repeats are anchored to both left and right flanks, in contrast to complex structure identified in PME duo. Shorter allele corresponds to TTTTA repeats of size 12. **(B)** Waterfall plot from Nanopore of long-range PCR product shows large, pure TTGTA expansion at FAME3 locus for individual identified through WES screening.

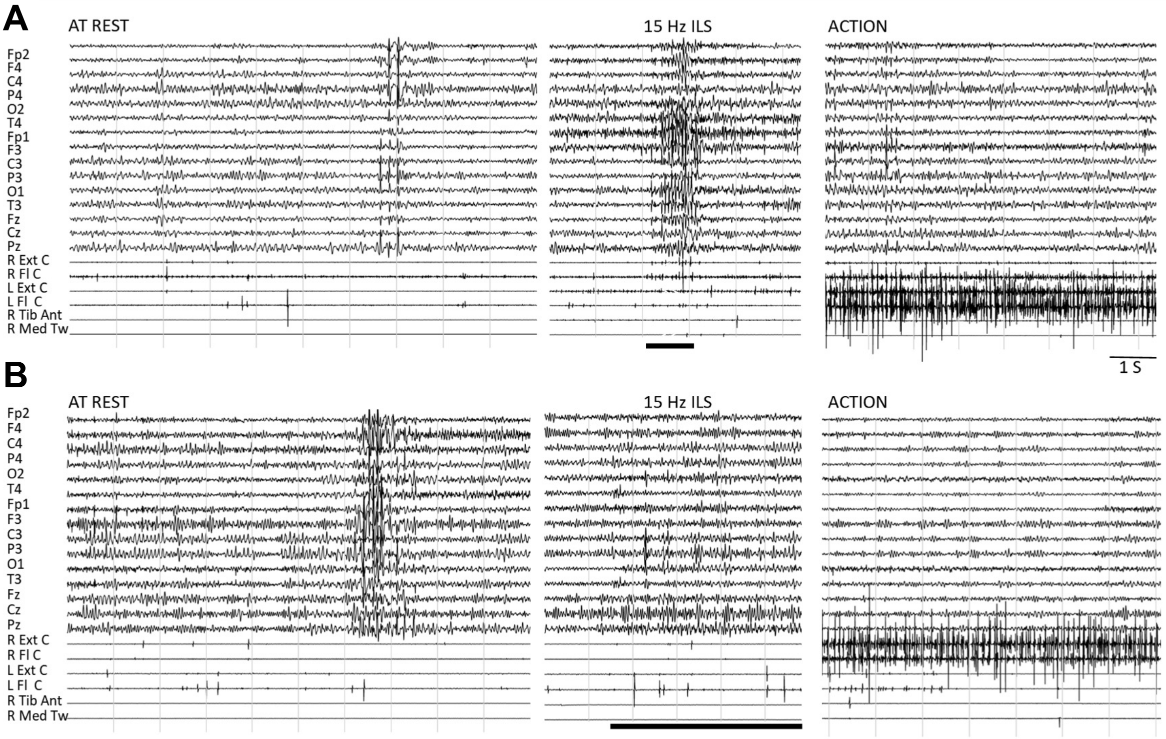

Supplementary Figure 3 EEG-EMG Polygraphy.

EEG-EMG recordings performed in **(A)** father (A-II-4) and **(B)** son (A-III-1). Top 15 channels denote EEG according to the international 10-20 system and bottom 6 channels show EMG (Ext C and Fl C = carpi extensor and flexor muscles; Tib Ant = tibialis anterior muscle; Med Tw = calf medial twin muscle). Time scale shown at right applies to all 6 panels. Left panels (at rest) show rare spontaneous short EEG paroxysms and brief independent EMG bursts. Middle panels show paroxysmal response to photic stimulation at 15 Hz Intermittent Light Stimulation (ILS); period of stimulation denoted by thick horizontal lines. Right panels (with action) show quasi-rhythmic myoclonus elicited by active movements.
